# Anemia prevalence and etiology among women, men, and children in Ethiopia: a study protocol for a national population-based survey

**DOI:** 10.1186/s12889-019-7647-7

**Published:** 2019-10-24

**Authors:** Amare Worku Tadesse, Elena C. Hemler, Christopher Andersen, Simone Passarelli, Alemayehu Worku, Christopher R. Sudfeld, Yemane Berhane, Wafaie W. Fawzi

**Affiliations:** 1 Department of Reproductive Health, Nutrition and Population, Addis Continental Institute of Public Health, Addis Ababa, Ethiopia; 2 Department of Global Health and Population, Harvard T.H. Chan School of Public Health, Boston, MA USA; 3 Department of Epidemiology, Harvard T.H. Chan School of Public Health, Boston, MA USA; 4 Department of Nutrition, Harvard T.H. Chan School of Public Health, Boston, MA USA; 5 Department of Epidemiology and Evaluation, Addis Continental Institute of Public Health, Addis Continental Institute of Public Health, Addis Ababa, Ethiopia

**Keywords:** Anemia, Etiology, Diet, Iron, Biomarkers, Ethiopia

## Abstract

**Background:**

Anemia remains a public health challenge in Ethiopia, affecting an estimated 56% of children under age 5 years, 23% of women of reproductive age and 18% of adult men. However, anemia etiology and the relative contribution of underlying risk factors for anemia remains unclear and has hindered implementation of anemia control programs.

**Methods/design:**

Anemia Etiology in Ethiopia (AnemEE) is a population-based cross-sectional survey of six regions of Ethiopia that includes children, women of reproductive age, and men from regionally representative households. The survey will include detailed assessment of anemia, iron, inflammatory and nutritional biomarkers, diet, comorbidities, and other factors. The objectives of AnemEE are 1) to generate evidence for decision-making on the etiology of anemia in Ethiopia among men, women and children and 2) to simulate the potential effect of iron fortification and other interventions on the prevalence of anemia and risk of iron overload.

**Discussion:**

AnemEE will provide the most comprehensive evaluation of anemia etiology in Ethiopia to date due to its detailed assessment of diet, biomarkers, infections and other risk factors in a population-based sample. By generating evidence and simulating potential interventions, AnemEE will inform the development of high-impact anemia control programs and policies.

**Trial registration:**

ClinicalTrials.gov, NCT04002466. Registered on 28 June 2019. Retrospectively registered.

## Background

Worldwide, anemia affects over 1.5 billion people, which is approximately a quarter of the global population [1]. Anemia, or low hemoglobin concentration, is known to negatively affect cognitive and motor function, increase the risk of maternal and child death, and cause fatigue and low work productivity [2–4]. Due to the wide range of adverse effects of anemia, the World Health Assembly (WHA) established a commitment to halve anemia prevalence from 2011 levels in women of reproductive age by 2025 [1]. The national prevalence of anemia in the 2016 Ethiopia Demographic and Health Survey (DHS) was estimated to be 56% among children under the age of 5 years, 23% among women of reproductive age (15–49) and 18% among adult men (15–49), with greater prevalence in rural areas and regions with large pastoralist communities [5]. Ethiopia is strongly committed to promoting health and wellbeing in the country, and anemia control is a priority. However, the relative contribution of the underlying causes of anemia in Ethiopia remains unclear.

The causes of anemia in low- and middle-income countries are multifactorial. It is estimated that half of anemia cases worldwide are attributable to iron deficiency; however, these reports have not assessed the relative contribution of iron deficiency and other risk factors at regional or country levels [6]. Non-iron deficiency causes of anemia include parasitic diseases and infections such as malaria [7], hookworm infections [8], and schistosomiasis [9]; other micronutrient deficiencies including folic acid, vitamin A, and vitamin B12 [10]; and genetic hemoglobinopathies such as sickle cell disease and thalassemia [11].

The 2013 Ethiopian National Food Consumption Survey (ENFCS) found that dietary iron intake at the population-level in Ethiopia was high, particularly among adult men and women [12]. As determined by a single 24-h dietary recall, only 12.9% of women of reproductive age and 3.2% of adult urban men had inadequate iron intake. In contrast, there was a high prevalence of excessive iron intake; 64% of women of reproductive age and 89% of adult urban males had excessive intakes based on World Health Organization recommended tolerable upper intake levels [13]. However, the ENFCS was focused on diet and therefore did not include biomarkers of anemia and iron. The 2016 Ethiopian National Micronutrient Survey (ENMS) directly assessed iron biomarkers and determined that the prevalence of iron deficiency anemia (IDA) as assessed by ferritin was 17.8% among children and 5.8% among non-pregnant adult women [14]. However, the ENMS lacked dietary intake data and did not include adult men in the survey. In addition, seasonality is likely an important determinant of iron intake and anemia prevalence in Ethiopia; a recent small longitudinal study of 216 pregnant women in rural Oromia and Tigray regions found that the prevalence of anemia increased from 21.8% during postharvest season to 40.9% in pre-harvest season [15]. The ENMS and ENCFS were conducted in single rounds and therefore were not able to examine seasonal variation in iron intake and anemia prevalence.

In order to address the current evidence gaps, we are undertaking a population-based cross-sectional survey to evaluate anemia prevalence and etiology in Ethiopia. The Anemia Etiology in Ethiopia (AnemEE) study will assess anemia, dietary intake, iron and other nutritional biomarkers, soil-transmitted helminth infections, malaria, and inflammatory biomarkers among children, women of reproductive age, and men in six regions of Ethiopia. We intend to determine the magnitude and relative contributions of the underlying causes of anemia and simulate potential interventions, in order to guide high-impact and safe anemia control intervention programs and policies.

## Methods/design

AnemEE is a population-based cross-sectional survey of children, adult females of reproductive age and adult males in six regions of Ethiopia, carried out in two seasons. The AnemEE protocol was developed by collaborators at the Harvard T.H. Chan School of Public Health in the United States and Addis Continental Institute of Public Health in Ethiopia. The first participant was enrolled in the study on January 27, 2019 and data collection is expected to continue through August 2019.

### Primary objectives

The overall goal of AnemEE is to generate evidence on the etiology of anemia in adults and children in Ethiopia to inform anemia control efforts. The two primary aims of the study are to (a) determine the prevalence and relative contribution of risk factors for anemia, iron deficiency anemia, and potential iron overload among children aged 6–59 months, adult women of reproductive age (15–49 years) and adult men (15–49 years) in six regions of Ethiopia and (b) simulate the potential effect of iron fortification, improved sanitation, deworming and other interventions on anemia, IDA, and iron overload among children and adult men and women.

### Study population

AnemEE will be conducted in six regions of Ethiopia: Amhara, Tigray, Oromia, Addis Ababa, Afar and Southern Nations, Nationalities, and Peoples’ Region (SNNPR). These regions were selected due to differences in their agroecology, anemia prevalence, and potential for iron overload. In addition, these regions cover a range of agrarian, urban/rural and pastoralist communities. Children (aged 6–59 months) and adult women of reproductive age (15–49 years) are included in the study population due to known high risk of anemia and poor health outcomes. Adult men are included as iron overload is a concern if an iron fortification intervention were to be implemented. Due to differences in diet between seasons in Ethiopia, the survey will be repeated in 2 rounds; the first during the short Belg season (dry season February–April) and the second in the major Meher season (wet season May–September). Both survey rounds will be conducted in the same kebeles (the smallest administrative units in Ethiopia), but the second round will be conducted in a different quadrant of each kebele so that each survey includes different individuals.

**Fig. 1:**
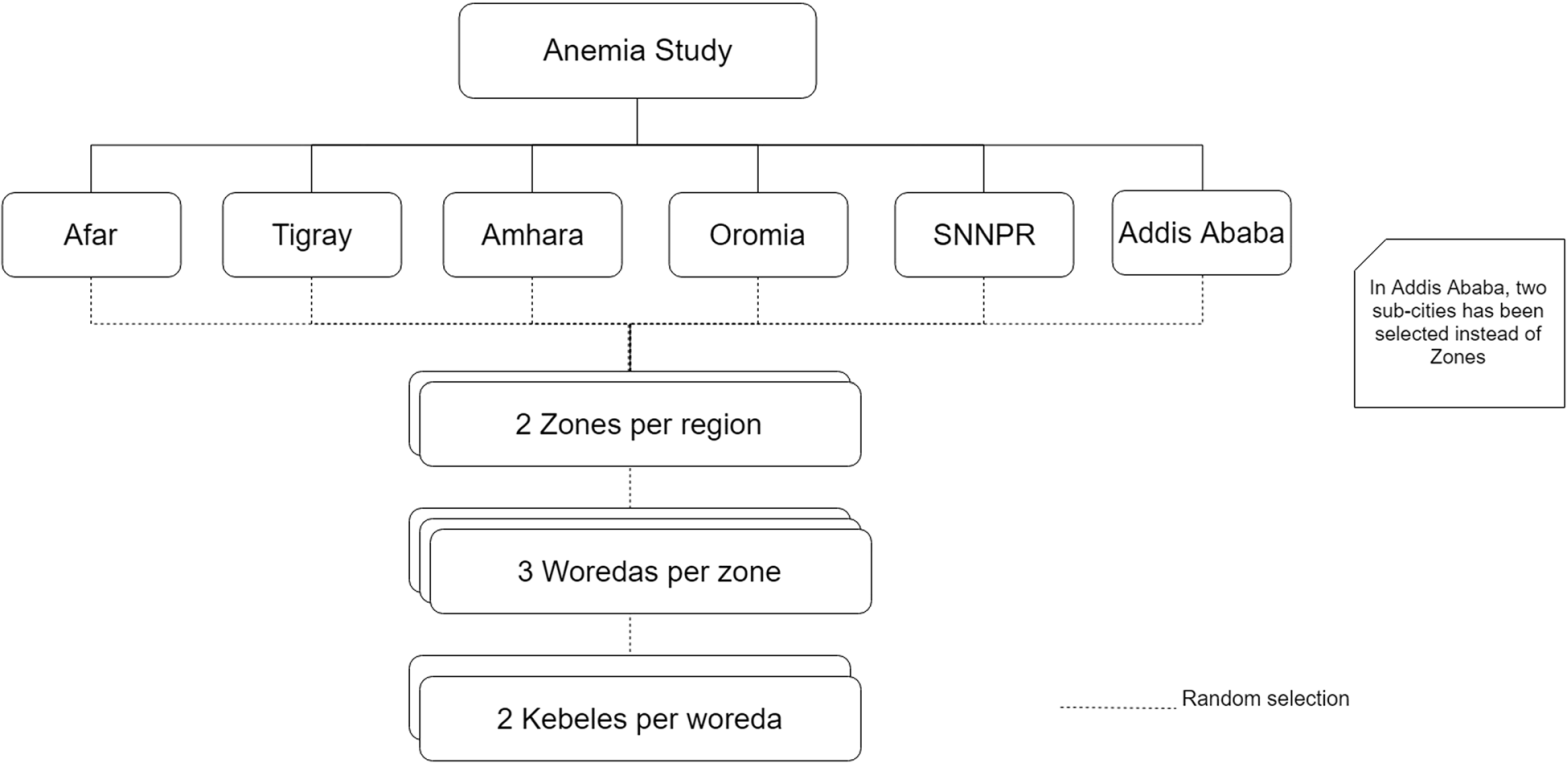
Sampling approach for the Anemia Etiology in Ethiopia (AnemEE) survey

**Fig. 2:**
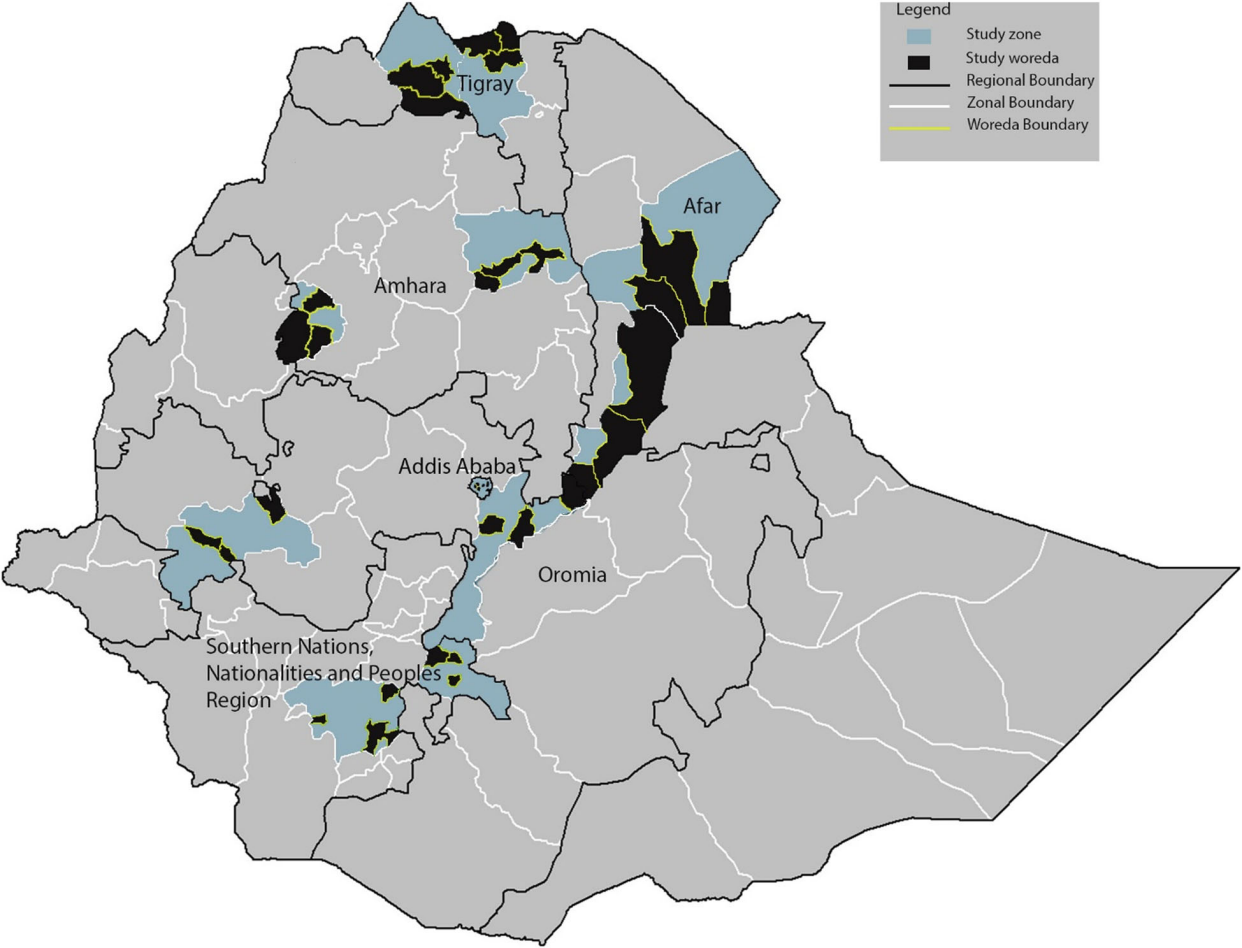
Zones and woredas included in the Anemia Etiology in Ethiopia (AnemEE) survey (Source: created by authors)

A stratified two-stage sampling method will be used to obtain regionally representative samples of urban and rural children and adult women and men in each region in each of the two seasons **(**Fig. 1**)**. In the first stage, two zones from each region will be randomly selected, and within each zone, 3 woredas (districts that collectively make up a zone) will be selected **(**Fig. 2**).** Within each woreda, administrative data from the regional and district administrative offices will be used to randomly sample 2 kebeles (12 kebeles total per region). In the second stage of sampling, each kebele will be divided into quadrants and a central zone, and one of these five areas will be randomly selected. Within each selected area, random households will be visited until 30 eligible households are identified, which will each include at least one woman of reproductive age. Of these, a simple random sample of 17 households per kebele will be selected for participation in the study using computer generated random numbers. Of the 17 households in each kebele, 17 women aged 15–49 years (one per household), 10 children aged 6 to 59 months (no more than one per household) and 7 men aged 15–49 years (no more than one per household) will be included in the study.

### Sample size

We calculated the minimum sample sizes required for each population group by region assuming the anemia prevalence determined in the 2016 Ethiopia DHS [5] by age, sex and region, 10% precision overall, 90% participation at the household, 90% participation at the individual level, and a design effect of 2.0. Between the two independent population level surveys, in the short Belg season (February–April) and in the major Meher season (May–September), a sample size of 4896 participants was calculated to provide regionally representative anemia estimates for children, adult women, and adult men in the six selected regions. We increased the total sample size to 5100 to increase precision.

### Data collection

Field and specimen collection standard operating procedures were developed and piloted in one urban and one rural kebele. Training manuals including a comprehensive description of all field work and data collection procedures were used to train all study staff. Enumerators with experience in large-scale nutrition-related surveys were recruited and trained on the study procedures. Experienced phlebotomists were recruited and trained on specimen collection and processing in the field. A supervision plan was developed to maximize data quality.

In each survey, trained enumerators will visit participating households selected during the sampling process to enroll participants and obtain informed consent. For each participant, the enumerators will seek written informed consent for a blood draw to assess serum biomarkers of anemia and a stool specimen to screen for soil-transmitted helminth infections. After enrolling participants on the first visit, the enumerators will return on a separate occasion to administer the questionnaires and collect biological specimens from the woman selected from that household and also the man or child (if applicable for that household). If one of the study participants is not available at the time of interview, the enumerator will return to the household up to three times to complete the assessments and collect the biological specimens. If the participant is still not available after three visits, that participant will be excluded. Table 1 provides an overview of data that we plan to collect for each man, woman and child. All questionnaires and dietary assessments will be administered on tablets using an electronic data collection platform, SurveyCTO (developed by Dobility, Inc.). During the survey, data will be continuously uploaded and monitored for quality.

**Table 1:** Questionnaire and biological assessments administered to women, children and men in the Anemia Etiology in Ethiopia (AnemEE) survey

Assessments	Women of reproductive age (15–49 years)	Children^a^ (6–59 months)	Adult Men (15–49 years)
Education, employment and other sociodemographic characteristics	X		X
Water, sanitation and hygiene	X		
Household characteristics	X		
Food fortification	X		
Pregnancy	X		
Morbidity in past 2 weeks	X	X	X
Household food sources	X		
24 h recall	X	X	X
Food frequency questionnaire	X	X	
Anthropometry (weight, height, MUAC)	X	X	X
Blood pressure	X		X
Hemoglobin	X	X	X
Malaria rapid diagnostic test	X	X	X
Soil-transmitted helminths	X	X	X
Ferritin^b^	X	X	X
C-reactive protein^b^	X	X	X
Folate^b^	X	X	X
Vitamin B-12^b^	X	X	X

^a^questionnaires completed on behalf of the child by the child’s primary caregiver

^b^We will attempt to obtain serum from all men, all children and a subset of women (approximately 50%)

#### Demographic, environmental, and socioeconomic indicators

Questionnaires were developed with input from stakeholders to assess individual and household demographic and economic indicators including (but not limited to): age, education, water source and quality, sanitation, employment, household assets, birth history, khat use (chewing of leaves to produce amphetamine-like effects), smoking status, pregnancy status, and other potential demographic, environmental and socioeconomic determinants of anemia and dietary iron intake. These instruments include questions from the Ethiopia 2016 DHS survey, which will allow for direct comparisons.

#### Dietary assessment

For all children, adult female and adult male participants, dietary intake will be measured with both a 24-h recall and a food frequency questionnaire (FFQ) with a 7-day recall. Based on guidelines described by Gibson [16], we developed a 24-h recall tailored to the unique cultural and dietary characteristics of Ethiopia, and pilot tested it in one urban and one rural kebele. The 24-h recall will be administered in multiple passes, which was shown to maximize recall accuracy for quantification [17]. In the first pass, the respondent reports all food and drink intake for the preceding day. In the second pass, the interviewer probes for details on the time, type and quantity of food or drink consumed. In the third pass, the interviewer asks for detailed recipes and in the final pass, the interviewer reviews all food reported in order, prompting for commonly unreported items, and clarifying uncertainties. In order to calculate within-person variability in dietary intake, in one kebele per region (in each survey), all participants will complete two 24-h recalls, one at the beginning of the survey round and another at the end of survey round. The target number of men, women and children with repeated measurements is 400. For the 24-h recall, Ethiopian food composition tables [18] will be used to calculate nutrient intakes. In cases where certain food items and nutrients are not available in the Ethiopian tables, food composition databases from other countries will be used to supplement the Ethiopian tables.

Dietary intake during the past 7 days will be measured using a food frequency questionnaire (FFQ) for all participants. Participants will be asked a series of questions to determine the number and timing of fasting periods within the previous week, as this may also affect intake of particular nutrients and anemia status. We developed the FFQ based on the food items in the Ethiopian food composition tables and piloted the tool in one urban and one rural kebele. For the FFQ, Ethiopian food composition tables will be used to calculate nutrient intakes for all participants, again supplementing with other food composition tables when necessary [18].

#### Portion size estimation

In designing the 24-h recall and FFQ instruments for this population, we estimated portion size for traditional Ethiopian foods using local reference sizes where appropriate and adapting portion sizes to the specifications of each traditional food. For example, for traditional breads such as a grain-based flatbread known as ‘injera,’ portion size is estimated using counts of folds and fractions of a whole injera. Mixed dishes, such as sauces and stews, are estimated by volume using standard household cooking measures displayed as photographs of bowls and spoons, which are representative of the range of portion sizes commonly consumed in Ethiopia. For whole foods and recipe ingredients such as fruits, vegetables and meats, pictures of different sizes of the food item are displayed as portion size options. Three to five sizes of each food are displayed based on the variety of sizes available at local markets. Photographs of the cooking/serving utensils (bowls, spoons, cups, etc.) and whole foods were taken using standardized methodology and including reference objects to maximize accuracy of portion size estimation based on the photographs [19]. For analysis of both the 24-h recall and FFQ, the reported portion size (estimated volume or number of food items consumed) will be converted into grams, for use in calculation of nutrient intake.

#### Anthropometry

Height (or length for children under 2 years of age), weight, and mid-upper arm circumference (MUAC) will be measured for all participating children, women and men. All measurements will be collected in triplicate and recorded electronically on the tablets. Child height/length-for-age (HAZ/LAZ), weight-for-height/length (WLZ/WHZ), and weight-for-age (WAZ) z-scores will be calculated using the WHO child growth 2006 standards [20]. Body mass index will be calculated for adult men and women.

#### Blood and stool collection

Trained field workers will collect venous blood and stool at the household from all women of reproductive age, men and children included in the survey. Two mL of blood will be collected from all men, women, and children in a lavender top vacutainer containing the anticoagulant Ethylenediaminetetraacetic acid (EDTA) for hemoglobin level measurements, malaria rapid diagnostic test (RDT), and dried blood spots. Five mL of blood will be collected in a red top vacutainer (without anticoagulant) from all men (with a target of 7 men per kebele), all children (with a target of 10 children per kebele) and a subset of women of reproductive age (with a target of 9 out of the 17 women participating in the study per kebele). Blood samples will be transported in an ice box from the household to a mobile lab where red top vacutainer samples will be centrifuged and aliquoted to store serum in cryovials within 2 hours and 30 min of collection. Serum samples will be stored in a portable − 20 °C freezer and will be transported to a central regional lab within 24 h. Samples will be shipped to International Clinical Laboratories (ICL) in Addis Ababa once per week for assessment of biomarkers and subsequent storage at − 80 °C. Stool samples will be preserved with 10% formalin within 2 hours of collection. These methods are consistent with methods used in the ENMS [14].

#### Hemoglobin and biomarkers assessment

For all participants, venous blood from lavender-top EDTA tubes will be drawn to assess hemoglobin concentration using a Hemocue HB 201+ analyzer. Hemoglobin levels will be adjusted for altitude, sex, and smoking status to determine anemia status [21]. All participants found to be anemic will be referred to a Health Extension Worker and health center for management that adheres to Ethiopian national guidelines. Stored serum samples will be tested for ferritin, C-reactive protein (CRP), folate and vitamin B12 by ICL in Addis Ababa.

#### Malaria, soil-transmitted helminth and morbidity assessment

The presence of malaria parasites will be assessed by a RDT from lavender-top EDTA tubes. All participants with parasitemia will be referred to the Health Extension Worker and health center for management that adheres to Ethiopian national guidelines. All participants will be asked about the presence and frequency of diarrhea, fever, vomiting, difficulty breathing and signs and symptoms of morbidities in the last 2 weeks. We will also collect information on any hospitalizations and medications taken in the last 2 weeks.

Stool samples will be collected from all children, adult females, and adult males. For each stool specimen, two Kato–Katz slides will be prepared and examined by microscopy for Ancylostoma duodenale (hookworm), Ascaris lumbricoides (roundworm), and Trichuris trichiura (whipworm) ova to determine the presence and intensity of infection.

### Statistical analysis

Anemia will be defined by standard hemoglobin definitions by sex and age (Hb < 11 g/dL for children; < 12 g/dL for non-pregnant adult women; < 13 g/dL for adult men). All hemoglobin levels will be adjusted for altitude, sex, and smoking status [21]. Ferritin will be used to assess iron status. All ferritin levels will be corrected for inflammation using the Biomarkers Reflecting Inflammation and Nutritional Determinants of Anemia (BRINDA) regression correction method [22]. We also will examine the prevalence of iron excess using adjusted serum ferritin concentrations [23]. All prevalence estimates will be calculated by region, urban/rural residence, sex, season, and age and take into account the sampling design.

Risk factors for anemia will be examined for anemia, IDA, and excess iron using generalized linear models taking into account the sampling design and clustering to calculate relative risks [24]. Potential differences in magnitude of risk (effect modification) by age, sex, gender, season, region, urban/rural residence and other factors of interest will be examined.

Population attributable risk percent (PAR%) will be calculated for each risk factor to estimate the percentage of anemia cases that can be independently attributed to that specific risk factor in the population [25, 26]. The PAR% will be used to estimate the percentage of anemia cases that would have been avoided in our study population if a specific risk factor was eliminated. These PAR% estimates will be useful for prioritization in the design of anemia control programs, as they take into account both the prevalence of risk factors and the magnitude of the risk for anemia.

## Discussion

Anemia remains a public health challenge in Ethiopia and prevention and evidence-based treatment strategies are urgently needed. However, there is a lack of information on anemia etiology, hindering the development of evidence-based anemia control efforts. To address the current evidence gaps, we designed the AnemEE survey to evaluate anemia etiology in Ethiopia. This cross-sectional survey in six regions of Ethiopia includes detailed assessment of usual dietary intake through the use of both a 24-h recall and FFQ, anemia and iron biomarkers, soil-transmitted helminth infections, malaria, and inflammatory biomarkers among a population representative sample of men, women, and children. These data are intended to determine the relative contributions of the underlying causes of anemia in order to guide high-impact and safe anemia control intervention programs and policies. To provide further guidance, we will also simulate the impact of introduction of iron fortification and other potential interventions on anemia and iron overload.

Although previous population-based studies in Ethiopia have assessed anemia, AnemEE is unique in that it assesses both anemia and risk factors simultaneously, enabling us to examine anemia etiology. AnemEE will also give us the ability to investigate the effect of seasonality on iron intake and anemia prevalence. Additionally, AnemEE has both consumption data and biological markers of anemia and iron status, which will allow us to estimate the effect of simulated dietary interventions on anemia and iron levels. The ENFCS simulations predicted that excessive iron intake would occur in adults and children in almost all regions if universal fortification of flours or oils were implemented, but this survey only had data on consumption and as a result could not model effects on anemia and excess iron [12]. Furthermore, we will also examine the effects of other health interventions such as deworming and improved sanitation, on reducing the prevalence of anemia and the risk of iron overload.

In light of the limited dietary assessment tools available for Ethiopia, the dietary assessment methods used in AnemEE are a strength of the study, and documenting the operational process of developing and implementing these methods can inform future studies and efforts to advance dietary assessment in Ethiopia. In order to formulate evidence-based nutritional strategies and policies aimed at ensuring adequate nutrient intake, evaluation and validation of reliable dietary assessment methods is important. Although several methodologies to measure dietary data exist, they have had variable success to collect valid dietary data and there is very limited information on their use in Ethiopia [27–31]. Additionally, there are currently no peer-reviewed publications from large-scale surveys in Ethiopia to enable replication of the methods used for dietary assessment. Thus, standardized measures to collect valid dietary data and biomarkers are needed to address the gaps in measuring dietary nutrient intake in Ethiopia. The electronic data collection system and portion size estimation methods used in AnemEE are novel in this context, and can be used to strengthen dietary assessment in future studies.

AnemEE will provide the most comprehensive evaluation of anemia etiology in Ethiopia to date, which will contribute to the evidence base and fill current knowledge gaps regarding prevalence and causes of anemia in Ethiopia. Currently, there is not sufficient evidence to develop high-impact and safe anemia control efforts; therefore, generating data on anemia etiology and simulating potential interventions is critical to inform anemia control programs and policies in Ethiopia.

### Study status

Study enrollment started on January 27, 2019 and recruitment is ongoing as of August 06, 2019.

## Data Availability

Not applicable.

## Abbreviations

AnemEE: Anemia Etiology in Ethiopia study
BRINDA: Biomarkers Reflecting Inflammation and Nutritional Determinants of Anemia (
CRP: C-reactive protein
DHS: Demographic and Health Survey
EDTA: Ethylenediaminetetraacetic acid
ENFCS: Ethiopian National Food Consumption Survey
ENMS: Ethiopian National Micronutrient Survey
FFQ: Food frequency questionnaire
ICL: International Clinical Laboratories
IDA: Iron deficiency anemia
PAR%: Population attributable risk percent
RDT: Rapid Diagnostic Test
SNNPR: Southern Nations, Nationalities, and Peoples’ Region
WHA: World Health Assembly

## References

[1] World Health Organization (2014). Comprehensive implementation plan on maternal, infant and young child nutrition.

[2] Balarajan Y, Ramakrishnan U, Ozaltin E, Shankar AH, Subramanian SV (2011). Anaemia in low-income and middle-income countries. Lancet.

[3] Haas JD, Brownlie T (2001). Iron deficiency and reduced work capacity: a critical review of the research to determine a causal relationship. J Nutr.

[4] Haider BA, Olofin I, Wang M, Spiegelman D, Ezzati M, Fawzi WW (2013). Anaemia, prenatal iron use, and risk of adverse pregnancy outcomes: systematic review and meta-analysis. BMJ (Clinical research ed).

[5] Central Statistical Agency Ethiopia and ICF International (2016). Ethiopia demographic and health survey 2016: key indicators report.

[6] Murray CJ, Ezzati M, Lopez AD, Rodgers A (2003). Comparative quantification of health risks conceptual framework and methodological issues. Population Health Metr.

[7] Korenromp EL, Armstrong-Schellenberg JR, Williams BG, Nahlen BL, Snow RW (2004). Impact of malaria control on childhood anaemia in Africa -- a quantitative review. Tropical Med Int Health.

[8] Smith JL, Brooker S (2010). Impact of hookworm infection and deworming on anaemia in non-pregnant populations: a systematic review. Tropical Med Int Health.

[9] King CH, Dickman K, Tisch DJ (2005). Reassessment of the cost of chronic helmintic infection: a meta-analysis of disability-related outcomes in endemic schistosomiasis. Lancet.

[10] Bhutta ZA, Ahmed T, Black RE, Cousens S, Dewey K, Giugliani E (2008). What works? Interventions for maternal and child undernutrition and survival. Lancet.

[11] Weatherall DJ, Clegg JB (2001). Inherited haemoglobin disorders: an increasing global health problem. Bull World Health Organ.

[12] Ethiopian Public Health Institute (2013). Ethiopian national food consumption survey.

[13] Allen L, de Benoist B, Dary O, Hurrell R, World Health Organization, Food and Agriculture Organization of the United Nations (2006). Dietary reference values: Estimated Average Requirements, Recommended Nutrient Intakes and upper limits. Guidelines on food fortification with micronutrients.

[14] Ethiopian Public Health Institute (2016). Ethiopian national micronutrient survey report.

[15] Roba KT, O'Connor TP, Belachew T, O'Brien NM (2015). Seasonal variation in nutritional status and anemia among lactating mothers in two agro-ecological zones of rural Ethiopia: a longitudinal study. Nutrition.

[16] Gibson RS, Ferguson EL (2008). An interactive 24-hour recall for assessing the adequacy of iron and zinc intakes in developing countries. International Food Policy Research Institute (IFPRI) and International Centre for Tropical Agriculture (CIAT).

[17] Ma Y, Olendzki BC, Pagoto SL, Hurley TG, Magner RP, Ockene IS (2009). Number of 24-hour diet recalls needed to estimate energy intake. Ann Epidemiol.

[18] Ethiopian Health and Nutrition Research Institute (1998). Food Composition Table For Use in Ethiopia. Addis Ababa, Ethiopia: Ethiopian Health and Nutrition Research Institute.

[19] Lazarte CE, Encinas ME, Alegre C, Granfeldt Y (2012). Validation of digital photographs, as a tool in 24-h recall, for the improvement of dietary assessment among rural populations in developing countries. Nutr J.

[20] Onis M (2006). WHO child growth standards based on length/height, weight and age. Acta Paediatr.

[21] World Health Organization (2001). Iron Deficiency Anaemia: Assessment, Prevention, and Control: A guide for programme managers.

[22] Namaste SM, Rohner F, Huang J, Bhushan NL, Flores-Ayala R, Kupka R (2017). Adjusting ferritin concentrations for inflammation: Biomarkers Reflecting Inflammation and Nutritional Determinants of Anemia (BRINDA) project. Am J Clin Nutr.

[23] World Health Organization (2011). Serum ferritin concentrations for the assessment of iron status and iron deficiency in populations.

[24] Zou G (2004). A modified Poisson regression approach to prospective studies with binary data. Am J Epidemiol.

[25] Eide GE, Gefeller O. Sequential and average attributable fractions as aids in the selection of preventive strategies. J Clin Epidemiol. 1995;48(5):645–55. 10.1016/0895-4356(94)00161-i 7730921

[26] Rückinger S, von Kries R, Toschke AM. An illustration of and programs estimating attributable fractions in large scale surveys considering multiple risk factors. BMC Med Res. 2009;9(1):7. 10.1186/1471-2288-9-7 PMC2636839 19166593

[27] Jariseta ZR, Dary O, Fiedler JL, Franklin N (2012). Comparison of estimates of the nutrient density of the diet of women and children in Uganda by household consumption and expenditures surveys (HCES) and 24-hour recall. Food Nutr Bull.

[28] Kigutha HN (1997). Assessment of dietary intake in rural communities in Africa: experiences in Kenya. Am J Clin Nutr.

[29] Amare B, Moges B, Moges F, Fantahun B, Admassu M, Mulu A (2012). Nutritional status and dietary intake of urban residents in Gondar. Northwest Ethiopia BMC Public Health.

[30] Lin CA, Boslaugh S, Ciliberto HM, Maleta K, Ashorn P, Briend A (2007). A prospective assessment of food and nutrient intake in a population of Malawian children at risk for kwashiorkor. J Pediatr Gastroenterol Nutr.

[31] Alemayehu AA, Abebe Y, Gibson RS (2011). A 24-h recall does not provide a valid estimate of absolute nutrient intakes for rural women in southern Ethiopia. Nutrition.

